# Postoperative weakness and anesthetic-associated rhabdomyolysis in a pediatric patient: a case report and review of the literature

**DOI:** 10.1186/s13256-022-03629-8

**Published:** 2022-10-25

**Authors:** John Floridis, Ruth Barbour

**Affiliations:** 1 Gove District Hospital, Nhulunbuy, Northern Territory Australia; 2 Royal Darwin Hospital, Darwin, Northern Territory Australia

**Keywords:** Suxamethonium, Succinylcholine, Anesthesia-associated rhabdomyolysis, Postoperative weakness, Case report

## Abstract

**Background:**

Anesthesia-associated rhabdomyolysis is a rare complication of surgery that causes postoperative myalgia, weakness, and potential renal failure if not managed promptly. Predisposing conditions that may lead to this complication include muscular dystrophies and myopathies.

**Case presentation:**

This rare case describes a pediatric non-Indigenous Australian patient developing this complication, with no known predisposing risk factors, and no clear etiology. A 9-year-old child with a background of asthma underwent an elective removal of keloid scar on her chest wall. The procedure was brief and uncomplicated, with an uneventful induction of anesthesia. During the emergence period, she developed acutely raised airway pressures with bronchospasm and laryngospasm requiring the use of salbutamol and suxamethonium with good effect. In the initial postoperative period, the patient complained of generalized myalgia and muscle weakness and was unable to mobilize independently. There was transient recovery to normal function; however, a recurrence of symptoms the following day with associated myalgias warranted admission to hospital. She was found to have rhabdomyolysis that was managed conservatively with a full recovery of several weeks. She was thoroughly investigated for any underlying cause, including genetic testing for malignant hyperthermia susceptibility (she had a variant of unknown significance but was negative for the known genetic abnormalities that cause malignant hyperthermia).

**Conclusion:**

This case report demonstrates the importance of considering anesthesia-associated rhabdomyolysis as a differential for acute postoperative weakness, and outlines an investigative approach. To the best of our knowledge, it is the first case described in the pediatric literature to report biphasic progression of symptoms.

## Background

Rhabdomyolysis is a condition whereby myocyte injury occurs, releasing internal components into the systemic circulation [1]. There are a large number of causes, and patients may present with a wide spectrum of clinical signs. Mild symptoms include muscle cramping or fatigue. Severe symptoms include severe muscle pain, with profound weakness. Renal impairment through direct tubular injury is a concern and requires treatment and close monitoring [2].

This case report describes unanticipated anesthesia-associated rhabdomyolysis in a pediatric patient in the postoperative setting, which is rare. It illustrates the clinical syndrome that evolved, including the workup required to identify and exclude potential causes of acute weakness and myalgias postoperatively.

## Case presentation

A 40 kg 9-year-old non-Indigenous Australian female was scheduled for an elective removal of a large right anterior deltoid keloid scar. Her medical history was significant for mild asthma. She had no known allergies, no past surgical or anesthetic history, and no significant family medical history, including no previous general anesthetics in either parent. Medications included regular inhaled fluticasone and salmeterol, with intermittent inhaled salbutamol as required for breakthrough asthma symptoms. She was normally a well and very active child. In the days leading up to her scheduled procedure, she had an intermittent dry cough with no exercise limitation, dyspnea, or fevers. Her preoperative physical examination demonstrated a mild intermittent wheeze in the right anterior basal lung field that resolved with salbutamol administration. The remainder of the examination was unremarkable. The decision to proceed with general anesthesia was based on a thorough assessment, consideration of all factors, and shared decision making with the family. Two factors leading to a general, rather than regional, anesthesia technique were the size of the lesion (with possibility of local anesthetic toxicity) and a previous traumatic experience when she had direct infiltration of intralesional corticosteroid previously.

The patient received no other premedication and was cooperative with preoxygenation prior to an intravenous induction. She received 50 mg of intravenous propofol and 50 μg of intravenous fentanyl for induction, and was effectively manually ventilated with a circle system during the apneic period. A first-generation laryngeal mask airway (LMA) device was inserted without difficulty with an initial pressure control mode of ventilation. Anesthesia was maintained with sevoflurane, oxygen, and air (FiO_2_ 0.50), with continuous monitoring of end-tidal capnography, oxygen saturation, heart, and noninvasive blood pressure.

The surgical procedure took 30 minutes in the supine position and was uncomplicated. During the maintenance phase of anesthesia, there were no periods of hemodynamic instability, hypoxia, hypercarbia, muscle rigidity, impaired ventilation, or hyperthermia. There were also no obvious abnormalities seen on continuous cardiac telemetry.

On emergence from anesthesia, 100 mg of intravenous propofol was given following cessation of sevoflurane to facilitate removal of her LMA under deep anesthesia. The patient subsequently became very difficult to ventilate with high airway pressures, hypoxia, and poor air movement with reduced chest expansion. As an emergency maneuver, her anesthesia was deepened with a further 100 mg of propofol and ongoing manual ventilation attempted with sevoflurane 8% on high flows of 100% oxygen. As this did not improve her ventilation or oxygenation, 50 mg of intravenous suxamethonium was administered with a rapid improvement in ventilation, oxygenation, and airway pressures. Given the rapidity of the situation, the differentials included laryngospasm or bronchospasm with interventions treating both possibilities. Upon recovery of oxygen saturation, chest auscultation demonstrated widespread wheeze initially, treated with in-line salbutamol, which was titrated to effect. She received approximately 1500 μg of inhaled salbutamol over a 15-minute period. She was supported with positive pressure mask ventilation until safe emergence from anesthesia, then transferred to the pediatric postoperative care unit.

Forty-five minutes later in the recovery bay, once completely awake, the patient described right neck pain and lower back pain that prompted a medical review by the anesthetic team. She had a benign cervical spine and musculoskeletal examination, with no red flags. At this stage, reassurance was provided.

Two hours later, in the discharge lounge, nursing staff noted an abnormal gait of the patient as she attempted to mobilize to the toilet. On further questioning, the patient was unable to walk unaided and needed full support to stand. A repeat medical review was requested, whereby she was found to have profound proximal lower limb muscle weakness, left lateral thigh pain, and right medial leg pain. She had reduced knee reflexes bilaterally and a broad-based unsteady gait. Her sensory function, upper limb examination, and cranial nerve examination were completely unremarkable.

To exclude salbutamol-induced hypokalemia as a possible cause of acute weakness, a venous blood gas was ordered, revealing a mild hypokalemia of 3.3 mmol/L. Having excluded this as a cause, the working diagnosis was suxamethonium-induced myalgia. She was kept for prolonged observation for 3 hours, until her symptoms improved. On discharge, she was able to mobilize unaided. She was provided with strict return precautions if her symptoms deteriorated.

The following day, her proximal muscle weakness had returned to involve all four limbs with associated tenderness. She was brought into the emergency department, needing assistance to mobilize. Urgent blood tests revealed a creatinine kinase (CK) of 22,679 units/L, suggestive of rhabdomyolysis. Her renal function, electrolytes, and urinalysis were normal. She was admitted for observation with conservative management and resolution of her CK levels over a 4-day period. She had a gradual return to full muscle strength and function over a period of 4 weeks.

An anesthetic alert letter was created, addressed to anesthetic, pediatric, and primary care teams to manage this patient as malignant-hyperthermia (MH) susceptible until evidence suggested otherwise as sole rhabdomyolysis may reflect a spectrum of this disorder. Given the age of the patient, a muscle biopsy (gold standard test) to definitely identify MH susceptibility was not performed. Genetic tests were used as an alternative, and detected the following:

Gene: *RYR1*

Genomic location (hg19): chr19:38979901G>A

Variant: c.5632G>A

Zygosity: heterozygous

Classification: 3c—a genetic variant of uncertain significance (VUS) with low clinical relevance.

As is the case with genetic testing, this variant may become clinically significant if other cases are found in the future.

## Discussion

There are many causes of acute rhabdomyolysis, broadly differentiated into inherited and acquired conditions. Outside the perioperative setting, conditions such substance abuse, medication, and trauma form the majority of acquired causes [3]. Inherited conditions include muscular dystrophies and metabolic disorders.

There are indeed patients who may undergo a general anesthetic with the conditions mentioned above—in such cases, the anesthetic and surgery are planned to minimize the risk, or severity, of rhabdomyolysis.

In the pediatric literature, the incidence of anesthesia-associated rhabdomyolysis is rare, of which the majority of cases (more than 95%) were due to an attributable cause when investigated [4].

On this occasion, the two possible agents potentially responsible for rhabdomyolysis included suxamethonium and sevoflurane. It is difficult to ascertain clinically, and in research, whether rhabdomyolysis is simply a progression of expected myalgias from suxamethonium use, as this medication is rarely used in isolation, but often with other anesthetic agents such as sevoflurane. It was difficult to make that assumption in this particular patient, as she initially improved but later deteriorated in a biphasic manner.

In patients who develop unexpected rhabdomyolysis following an anesthetic, other differentials must be considered.

### Anesthesia-induced rhabdomyolysis (AIR)

AIR is a very rare condition, with unknown incidence, and incompletely understood [5]. The hallmark features of this condition include marked hyperkalemia, peaked T waves on electrocardiogram (ECG), acidosis, and elevated CK. Hyperkalemia can lead to cardiac arrhythmias, and cardiac arrest if not identified and treated promptly.

### Malignant hyperthermia (MH)

A thorough inspection of the anesthetic record is essential to take note of medications administered, and any physiological abnormalities noted during the anesthetic, including hyperthermia, hypercarbia, hypoxia, ECG changes, tachycardia, and muscle rigidity, all of which may be suggestive of MH. This genetic disorder was first described in 1960 after several unexplained deaths under anesthesia occurred within a family [6]. Common anesthetic agents implicated in this condition include halogenated volatile agents (for example, sevoflurane, desflurane) and the depolarizing muscle-relaxant suxamethonium [5]. Importantly, patients do not need to exhibit all of these signs to be diagnosed with MH susceptibility. Delayed and isolated rhabdomyolysis in the postoperative setting is a recognized phenomenon, and has been reported in case reports, whereby patients have later been confirmed to be MH susceptible [7]. Furthermore, dedicated MH laboratories across the world keep a database of such variants in presentation. Testing for malignant hyperthermia susceptibility is specialized and undertaken by dedicated laboratories; it involves a mixture of genetic tests and muscle biopsy testing [8].

### Surgical and position factors

In the immediate postoperative period, any surgical factors must be considered, for example, acute compartment syndrome following an orthopedic fixation. Patient positioning, including skin and tissue breakdown, should be excluded with an extensive skin and musculoskeletal assessment.

### Subclinical underlying conditions

Postoperative rhabdomyolysis may be the first presentation of a previously unknown disorder involving neuromuscular dysfunction. While this discovery may happen as part of pediatric anesthesia, it is possible for it to occur in adult populations who may have not received an anesthetic prior, or where the condition is of mild severity. Conditions include, but are not limited to: muscular dystrophies, thyroid dysfunction, glycogen storage disease, mitochondrial disease, and disorders in fatty acid and carbohydrate metabolism [9, 10].

### General approach

Prior to performing any targeted investigation, the treating clinician should take a detailed history, which may provide clues. This should include any previous episodes of muscle pain or weakness and details surrounding these episodes (that is, medications taken, diet changes, exercise, procedures, infection). For example, in patients with glycogen storage dysfunction such as McArdle’s disease, they may provide a reliable history of predictable pain after several minutes of intense exercise. Following a detailed history, a neurological and musculoskeletal examination must be performed looking for any longstanding myopathy, wasting, neurological deficits, or gait abnormalities which may be seen in muscular dystrophies [5].

## Conclusions

This case demonstrates the need to be aware of anesthesia-associated rhabdomyolysis as a rare, but important, entity in the perioperative setting. To the best of our knowledge, this is the only case reported in the literature to describe a biphasic progression of symptoms. Furthermore, it is one of a few pediatric cases where no clear underlying disorder has been identified in the postoperative period. The symptoms and signs of anesthesia-associated rhabdomyolysis overlap with several other disorders that require exclusion, including anesthesia-induced rhabdomyolysis (AIR), and malignant hyperthermia. Treatment is largely supportive, with close monitoring required to ensure a full recovery.

## Data Availability

Not applicable.

## Abbreviations

AIR: Anesthesia-induced rhabdomyolysis
LMA: Laryngeal mask airway
MH: Malignant hyperthermia
